# How prepared is Brazil to tackle the COVID-19 disease?

**DOI:** 10.7189/jogh.10.020321

**Published:** 2020-12

**Authors:** Rodrigo Martins Moreira, Alejandra Carolina Villa Montoya, Sara Line Silveria Araujo, Rafaela Aparecida Trindade, Dara da Cunha Oliveira, Guilherme de Oliveira Marinho

**Affiliations:** 1 Department of Environmental Engineering, Federal University of Rondônia, Brazil; 2 Faculty of Basic Sciences and Common Areas, CBATA Group (Applied basic sciences), Tecnológico de Antioquia, Medellín, Colombia

The emergence of new diseases such as Sars-Cov-2 is a reflection of the expansion of anthropic activities on natural ecosystems. Food insecurity is also a key problem that causes the ingestion of wild protein sources, causing imbalance and the contact of human populations with pathogens unknown by modern medicine and raising the importance of health care infrastructure [1].

The first case in Brazil was reported on February 25, 2020, in the city of São Paulo; however, to date the number of confirmed cases, until May 28, 2020 nationwide was 391 222 confirmed and of 24 512 deaths, which are underestimated values due to the lack of mass tests. As a result of the city of São Paulo being a hub for several national and international transport systems, COVID-19 reached the entire Brazilian states and South American countries.

Brazil is facing immense challenges with the arrival of COVID-19, since the vast territorial extension, high population density in some cities, wide variety of air, land and sea routes with connections to the whole world, and a health system with limited access to methodologies for virus detection and attention through intensive care [2,3] make it hard to control the epidemic, increase the number of people susceptible to infection, reduce the response capacity of medical attention and increase the risks of death.

The COVID-19 epidemic has negative effects as well at the economic level, due to the large number of hospitalizations, prolonged quarantines, and closure of cities and local and global transport systems, which consequently affects the production chain, international relations and social functioning. In this context, Brazil as a developing country is particularly threatened.

The lack of information about COVID-19 hinders the response capacity of health personnel, government entities and the susceptible population, leading to an exponential increase in the number of confirmed cases. In the national context, it is necessary to analyze the factors that affect the spread of the virus, its control and infrastructure to respond to this emergency in order to identify places of risk and create strategies that allow reducing the negative impact of COVID-19. Disease mapping allow to understand and predict disease risk, considering observed cases within small regions and estimating the effect in a bigger region, according to disease characteristics [4]. Geographic Information Systems (GIS) are key tools that make it possible to prepare, store, process, retrieve, analyze and present geographic information and make appropriate decisions of response to the epidemics, being used to identify strengths and weaknesses, and thus target economic and human efforts effectively and efficaciously.

In this context, this study aims to answer how prepared is Brazil to tackle the COVID-19 disease by applying the Moran’s Autocorrelation Index using GIS.

Confirmed cases and health equipment data were acquired from the Brazilian Ministry of Health's Tabnet DataSUS platform [5]. Vectorial data for the administrative boundaries were acquired from the Brazilian Institute of Geography and Statistics (2015).

To apply spatial analysis, a technique based on the Moran’s Autocorrelation Spatial Index was used, through exploratory analysis of geospatial data associated with area features, in which it was suggested as a possibility of non-spatial statistical measure of correlation [6,7]. Direct correlation is indicated by positive values, ranging from 0 to +1, and inverse correlation by values between -1 and 0, the negative ones. In which, for the purpose of obtaining the significance estimate, we try to relate the test statistic to the normal distribution or, as a method of realization without assumptions, the pseudo-significance test.

Thereby, we have:

(HH) high-high: Value above the mean for the unit and its neighbors, indicating the existence of clusters of high values of the analyzed variable;
  - low-low: Value below the mean for the unit and its neighbors, indicating the existence of clusters of low values of the analyzed variable;
(HL) high-low: Value above the mean for the unit and below the mean for its neighbors;
(LH) low-high: Value below the mean for the unit and above the mean for its neighbors.

**Figure 1:**
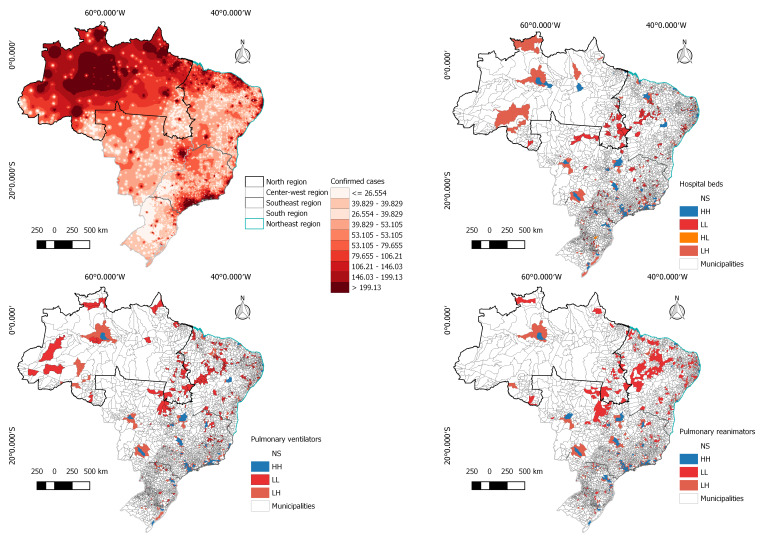
Key health infrastructure indicators to assess the preparedness of a country to tackle the Covid-19. Panel A. Confirmed cases distributed by regions (top left). Panel B. The spatial autocorrelation of pulmonary ventilators (top right). Panel C. Number of hospital beds (bottom left). Panel D. Number of pulmonary reanimators (bottom right). All figures are presented in municipal scale. NS – non significant, HH – High-High, LL – Low-Low, HL – High-Low, LH – Low-High.

Brazil is contained in South America and is the fifth largest country in territorial extension, the territory is divided into five regions, namely: North, Midwest, Northeast, Southeast and South, and is divided into 26 states and one Federal District, which results in 5570 municipalities. In Brazil, the estimated population is in the order of 210 million people [8] and a total of 391 222 confirmed cases for COVID-19 were registered from February 01th to May 25th, 2020. The results of the application of the Moran Index are presented in **Figure 1**.

The spread of the virus in the Brazilian territory may be linked to the financial market, since Brazilian foreign trade is fostered by China, which occupies the first place in the ranking of export recipients, so this is one of the countries that most sell to Brazil. Because of this evidence, China is the destination of many Brazilians.

Until May 28, 2020, southeast region was the most affected region, with 151 376- confirmed cases, the state of São Paulo was leading the number of confirmed COVID-19 cases, with approximately 89 483- infected people, and Rio de Janeiro a total of 42 398.

COVID-19 symptoms are varied, but part of the infected population needs hospital care. The gradual increase in positive cases in a short period suggests the need to supply health centers with personal protective equipment, beds, ventilators, pulmonary resuscitators, medications and other materials and equipment necessary for hospitalization, care and treatment of patients with more serious symptoms. Mattos et al. (2020) estimates that of the 20% of the population infected with SARS-CoV-2, 5% needed intensive therapy for 5 days [9]. From this scenario, it is assumed that of the 2 113 749 people sick with COVID-19 in Brazil, 422 750 will need hospital beds.

**Figure:**
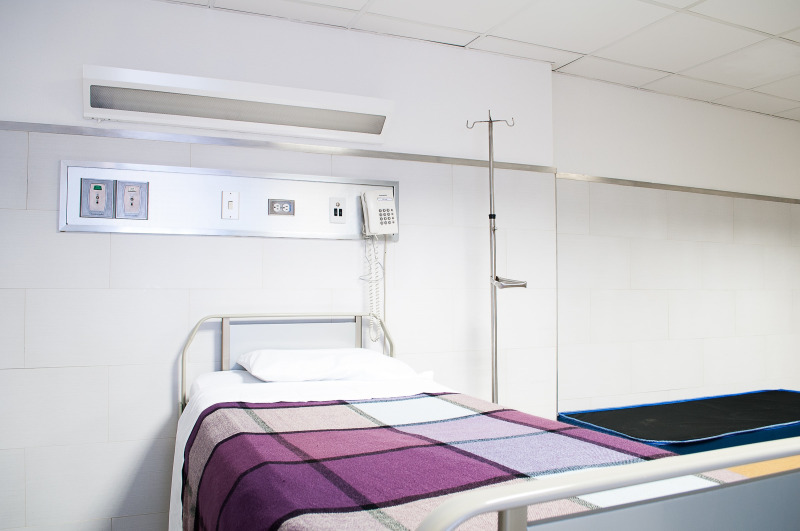
Photo: By Martha Dominguez de Gouveia on Unsplash.

The spatial autocorrelation of hospital beds in Brazil, presented values for the Global Moran Index of 0.13 (*P* > 0.01). Where 3.25% of municipalities present HH values, 4.44% present LL, 0.04% presented HL and 4.75% present LH values, as shown in **Figure 1**. HH clusters can be noticed in the southeast region. Specially around the municipality of São Paulo. Northeast and North region are present several clusters with LL and LH. In Brazil, these are the regions with lowest MHDI (municipal human development index) and highest Gini indexes, which clearly influences the health infrastructure.

Pulmonary ventilators are key for treating the COVID-19 patients. The spatial autocorrelation for pulmonary ventilators presented Global Moran Index values of 0.14 (*P* >0.01), presented 2.59% of municipalities with HH values, 5.27% of municipalities presented LL values and 4.87% presented LH values. Clusters can be noticed in southeast and south regions. Northeast and North regions have only 10% of municipalities with HH values.

The spatial autocorrelation for pulmonary reanimators presented Global Moran Index value of 0.14 (*P* > 0.01) with 2.80% of municipalities with HH values, 8.07% of municipalities with LL values and 4,. % of municipalities with LH values. The majority of LL values are clustered in northeast region.

These results raise the flag of years of lack of investments regard operational and human resources infrastructure and lack of political interest in the health public services in Brazil. The mapping and autocorrelation analysis are key for displaying discrepancies in the public health sector of Brazil. Health services requires revision and funding for universalization purposes [1].

The spatial autocorrelation made possible to answer how prepare is Brazil by analyzing the number of hospital beds, pulmonary ventilators and reanimators, factors that affect the dynamics of the pandemic and allow adequate decision making in the health sector. Spatial variability display that the northeast and southeast regions show less availability of health infrastructure, places where the number of people affected is enormous. North and Northeast regions are the most threatened by the COVID-19 disease regard lack of hospital infrastructure. This potentially leads to an imbalance between demand and supply, with precarious medical care for people with severe symptoms and increased deaths.

This work is key for assessing the relationship between the number of people infected and health care systems, allowing decision-makers to focus efforts. Measures such as the capacity for hospital care, isolation, closure of mass transport systems and hygiene are recommended to avoid collapse of the health system.
